# Targeting energy metabolism in brain cancer: review and hypothesis

**DOI:** 10.1186/1743-7075-2-30

**Published:** 2005-10-21

**Authors:** Thomas N Seyfried, Purna Mukherjee

**Affiliations:** 1 Biology Department, Boston College, Chestnut Hill, MA 02467, USA

**Keywords:** glioma, vascularity, caloric restriction, ketone bodies, metabolic control analysis, angiogenesis, apoptosis, inflammation, Warburg

## Abstract

Malignant brain tumors are a significant health problem in children and adults and are often unmanageable. As a metabolic disorder involving the dysregulation of glycolysis and respiration, malignant brain cancer is potentially manageable through changes in metabolic environment. A radically different approach to brain cancer management is proposed that combines metabolic control analysis with the evolutionarily conserved capacity of normal cells to survive extreme shifts in physiological environment. In contrast to malignant brain tumors that are largely dependent on glycolysis for energy, normal neurons and glia readily transition to ketone bodies (β-hydroxybutyrate) for energy *in vivo *when glucose levels are reduced. The bioenergetic transition from glucose to ketone bodies metabolically targets brain tumors through integrated anti-inflammatory, anti-angiogenic, and pro-apoptotic mechanisms. The approach focuses more on the genomic flexibility of normal cells than on the genomic defects of tumor cells and is supported from recent studies in orthotopic mouse brain tumor models and in human pediatric astrocytoma treated with dietary energy restriction and the ketogenic diet.

## Introduction

The world-wide incidence of malignant brain tumors may be increasing in both children and the elderly [1-4]. Regardless of these ominous findings, the standard therapies for malignant gliomas (surgical resection and radiation) are basically the same today as they have been for over five decades [5-7]. While these therapies may retard glioma growth over the short term, they can facilitate glioma recurrence and enhance growth rate over the longer term through alterations in morphogenetic fields [8,9]. Chemotherapy has little long-term benefit on most malignant gliomas and is often associated with adverse effects that diminish the length or quality of life [7,10]. The therapeutic targeting of brain tumor-associated mutations may also be problematic as most tumor mutations arise as epiphenomena of tissue disorganization and their relationship to causality is uncertain [8,11,12]. Despite modest gains in survival with temozolomide chemotherapy, few things are more certain in the brain tumor field than the impotence of most current therapies [2,7,13]. Hence, new approaches are needed that can provide long-term management of malignant brain tumors while permitting a decent quality of life.

## Metabolic Control Analysis

Metabolic control analysis evaluates the degree of flux in metabolic pathways and can be used to analyze and treat complex diseases [14-16]. The approach is based on findings that compensatory genetic and biochemical pathways regulate the bioenergetic potential of cells and ultimately the phenotype [14,15,17]. As rate-controlling enzymatic steps in biochemical pathways are dependent on the metabolic environment of the physiological system, the management of disease phenotype depends more on the flux of the entire system than on the expression of any specific gene or enzyme alone [16-19]. In other words, complex disease phenotypes can be managed through self-organizing networks that display system wide dynamics involving glycolysis and respiration. Global manipulations of these metabolic networks can restore orderly adaptive behavior to widely disordered states involving complex gene-environmental interactions [15,17,20,21].

As abnormal energy metabolism and biological chaos are characteristics of brain tumors [8,22-24], the general principles of metabolic control analysis can be effective for brain cancer management. This hypothesis is based on the known differences in energy metabolism between normal and neoplastic brain cells. As long as brain tumors are provided a physiological environment conducive for their glycolytic energy needs, they will survive; when this environment is restricted or abruptly changed, they will either growth arrest or perish. Here we describe how new therapeutic approaches, which lower circulating glucose and elevate ketone bodies (acetoacetate and β-hydroxybutyrate), target brain tumors while enhancing the metabolic efficiency of normal neurons and glia.

## Energy Metabolism in Normal Brain Cells

**Figure 1:**
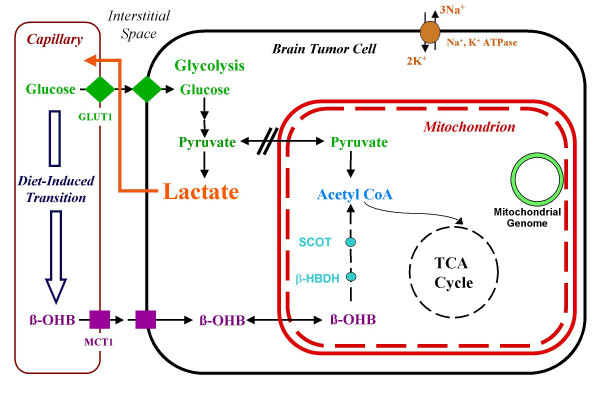
**Perspectives on the metabolic management of brain cancer through a dietary reduction of glucose and elevation of ketone bodies**. A dietary reduction in circulating glucose will increase ketone utilization for energy in normal neurons and glia. This will induce an energy transition from glycolysis to respiration. Cancer cells however, may be unable to transition from glucose to ketones due to alterations in mitochondrial structure or function (dashed lines). The double slash indicates a disconnection between glycolysis and respiration according to the Warburg hypothesis. Abbreviations: GLUT-1 (glucose transporter), MCT-1 (monocarboxylate transporter), SCOT (succinyl-CoA-acetoacetate-CoA transferase), β-OHB (β-hydroxybutyrate), β-HBDH (β-hydroxybutyrate dehydrogenase).

To manage brain cancer through metabolic targeting it is necessary to consider energy metabolism in the normal orthotopic tissue. Figure 1 illustrates some of the metabolic pathways discussed here. Under normal physiological conditions, the mature brain derives almost all of its energy from the aerobic oxidation of glucose [15,25,26]. The glucose transporter, GLUT-1, is enriched in the brain capillary endothelial cells and mediates the facilitated diffusion of glucose through the blood brain barrier. Most of the glucose is metabolized to pyruvate, which enters the mitochondria of neurons and glia and is converted to acetyl-CoA before entering the TCA cycle. Only about 13% of glycolytic pyruvate is converted to lactate under normal conditions [26]. Fatty acids are attached to lipoproteins and do not pass the blood brain barrier as fuel substrates, though octanoate may be an exception [26-28]. It is also unlikely that lactate is used for energy metabolism in adult brain, but this remains somewhat controversial [26,29-31]. Hence glucose is the primary, if not exclusive, brain metabolic fuel under normal physiological conditions.

While glucose is the preferred energy substrate, neurons and glia will metabolize ketones for energy under fasting-induced reductions of blood glucose. This is a conserved physiological adaptation to prolonged food restriction and evolved to enhance survival and maintain adequate brain function while sparing proteins [15,27,32-34]. Ketone bodies, consisting of acetoacetate, and β-hydroxybutyrate (β-OHB) are derived from fat catabolism in the liver and their concentration in blood is inversely related to that of glucose [21,35-37]. Ketone bodies are transported into the brain through the blood-brain barrier monocarboxylic transporters (MCTs), whose expression is regulated in part by circulating ketone and glucose levels [30,31,34,38-41]. β-OHB is the predominant blood ketone body and is rapidly oxidized to acetyl-CoA in the mitochondria through an enzymatic series involving 3-hydroxybutyrate dehydrogenase, SCOT (succinyl-CoA-acetoacetate-CoA transferase), and mitochondrial acetyl-CoA thiolase [33,34,42,43]. Acetone is a non-enzymatic byproduct of ketone body synthesis and is largely excreted in the urine or exhaled from the lungs [34,44].

Although the levels of glucose and ketones in brain are proportional to their levels in blood [34,45], the adult brain does not usually metabolize ketone bodies for energy unless blood glucose levels are reduced [36]. Therapeutic efficacy of the ketogenic diet is best when coupled to dietary energy restriction under which conditions circulating glucose levels are gradually reduced in conjunction with elevations of ketone bodies [15,21]. The emphasis here is on the term "gradual", as ketone bodies cannot be used for energy following acute hypoglycemia [46]. The situation is different, however, for *in vitro *preparations where neuronal glial interactions are disrupted and the blood brain barrier is absent [47,48]. The gradual transition from glucose to ketone bodies for energy *in vivo *requires a flexible genome for the coordinated integration of multiple metabolic networks according to principles of metabolic control analysis [15,36].

Ketone bodies are more energetically efficient than either pyruvate or fatty acids because they are more reduced (greater hydrogen/carbon ratio) than pyruvate and do not uncouple the mitochondrial proton gradient as occurs with fatty acid metabolism [14]. In contrast to glucose, ketone bodies by-pass cytoplasmic glycolysis and directly enter the mitochondria where they are oxidized to acetyl-CoA [44,49]. The amount of acetyl-CoA formed from ketone body metabolism is also greater than that formed from glucose metabolism [50]. This increases TCA cycle metabolites (from citrate to α-ketoglutarate) while reducing the mitochondrial NAD couple, [NAD^+^]/[NADH], and increasing the mitochondrial Q couple [Q]/[QH_2_] [14,50]. The difference between these couples increases the redox span between the NADH dehydrogenase complex (site I), and the CoQH_2_-cytochrome C reductase (site III) thus enhancing the mitochondrial proton gradient [14]. This enhances the energy available from the hydrolysis of ATP, ΔG'ATP, the cell's key energy reserve generated through the mitochondrial Fl ATPase [14,16]. Remarkably, the ketone body-induced increase in the ΔG'ATP is also accomplished using less oxygen [48,50]. These and other findings led Veech to designate ketone bodies as a "super fuel" [14].

## Ketones and Free Radicals

In addition to increasing ATP production while reducing oxygen consumption, ketone body metabolism can also reduce production of damaging free radicals [14,16,48]. The semiquinone of Q, the half reduced form, spontaneously reacts with oxygen and is the major source of mitochondrial free radical generation [14,51]. Oxidation of the Q couple reduces the amount of the semiquinone form thus decreasing superoxide production [14]. Since the cytosolic free NADP^+^/NADPH concentration couple is in near equilibrium with the glutathione couple, ketone body metabolism will increase the reduced form of glutathione thus facilitating destruction of hydrogen peroxide [14]. The reduction of free radicals through ketone body metabolism will also reduce tissue inflammation provoked by reactive oxygen species. Thus, ketone bodies are not only a more efficient metabolic fuel than glucose, but also possess anti-inflammatory potential.

## Energy Metabolism in Brain Tumors

In contrast to normal brain that oxidizes glucose as well as ketone bodies for energy, malignant brain tumors from either humans or animal models lack metabolic flexibility and are largely dependent on glucose for energy [21,23,52-58]. Enhanced glycolysis produces excess lactic acid that can return to the tumor as glucose through the Cori cycle [59] (Figure 1). Although some neural tumors metabolize ketone bodies, this metabolism is largely for lipid synthesis rather than for energy production [60,61]. Many tumors also have reduced activity of SCOT, the rate-controlling step for utilizing β-OHB as a respiratory fuel [42,62,63]. Consistent with these observations, we recently found that β-OHB could rescue normal mouse astrocytes under low glucose conditions, but could not rescue mouse astrocytoma cells [20]. Although glutamine may provide energy to some non-neural tumors, glutamine stimulates glycolysis in C6 rat glioma cells and may not serve as a direct respiratory fuel [64]. Hence brain tumors, like most malignant tumors, depend heavily on glucose and glycolysis for their metabolic energy and are generally unable to metabolize β-OHB for energy.

In addition to glycolytic dependence, most tumors including brain tumors, express abnormalities in the number and function of their mitochondria [65,66]. Such abnormalities would prevent the bioenergetic utilization of ketone bodies, which require functional mitochondria for their oxidation [47]. Warburg originally emphasized that the high glycolytic rate of tumors resulted from diminished or disturbed respiration [67,68]. While most cells die from damaged respiration, those cells that can enhance and modify their anaerobic glycolysis in response to respiratory damage will survive and form tumors [67,68]. Later studies in a variety of neural and non-neural tumor systems showed that these respiratory disturbances involve abnormalities in TCA cycle components, alterations in electron transport, and deficiencies in oxidative phosphorylation [23,55,69-71]. Mitochondrial DNA mutations, however, may not be involved [12]. Structural defects of the inner mitochondrial membrane, that would reduce or dissipate the proton motive gradient, could also prevent normal ATP production despite the appearance of oxidative metabolism, i.e., oxygen consumption and CO_2 _production [70,72,73]. Considered together, these findings indicate that brain tumors suffer from reduced respiratory capacity coupled to an increased glycolysis and lactic acid production.

Although persistent aerobic glycolysis (glycolysis in the presence of oxygen) or defects in the Pasteur effect (reduction of glycolysis in the presence of oxygen) are characteristics of many tumors, Warburg considered these phenomena too labile or too dependent on environmental conditions to be reliable indicators of tumor metabolism [68]. Rather, he emphasized the importance of defects in the coordination of glycolysis with respiration. The latency between tumor initiation and progression was considered the period necessary to disconnect respiration from glycolysis [68]. Recent studies in human glioblastoma cells suggest that this disconnect involves activation of the *Akt *oncogene rendering cancer cells dependent on aerobic glycolysis for continued growth and survival [74]. This is consistent with the Warburg hypothesis that the increased glycolysis of tumor cells occurs gradually in order to compensate for respiratory failure [68]. In contrast to normal brain cells, in which glycolysis and respiration are tightly coupled, tumor cells are defective in their ability to integrate energy metabolism between glycolysis and respiration [71]. It is these defects that will render brain tumor cells vulnerable to metabolic targeting through metabolic control analysis. Support for this possibility comes from studies with the ketogenic diet and dietary energy restriction.

## Dietary Energy and Brain Cancer

### The Ketogenic Diet

In 1995, Nebeling and coworkers attempted the first nutritional metabolic therapy for human malignant brain cancer using the ketogenic diet [75]. The ketogenic diet (KD) is a high fat low carbohydrate diet that has been used for decades as an effective therapy for refractory seizures in children [15,76,77]. The objective of the Nebeling study was to shift the prime substrate for energy metabolism from glucose to ketone bodies in order to disrupt tumor metabolism while maintaining the nutritional status of patients [75].

The patients in this landmark clinical study included two female children with nonresectable advanced grade brain tumors (anaplastic astrocytoma stage IV, and cerebellar astrocytoma stage III) [75]. Measurable tumor remained in both subjects following extensive radiation and chemotherapy. Although severe life threatening adverse effects occurred from the radiation and chemotherapy, both children responded remarkably well to the KD and experienced long-term tumor management without further chemo or radiation therapy [75]. Indeed, one of the patients is still alive and well at the time of this writing (Nebeling, personal communication). Positron Emission Tomography with fluro-deoxy-glucose (FDG-PET) also showed a 21.8% reduction in glucose uptake at the tumor site in both subjects on the KD [75].

Despite the logic of these studies and the dramatic findings, no further human studies or clinical trials have been conducted on the therapeutic efficacy of the KD for brain cancer. The reason for this is not clear but may reflect a preference of the major Brain Tumor Consortia for using "hand-me-down" drug therapies from other cancer studies rather than exploring more effective biological or non-chemotherapeutic approaches [7]. This is unfortunate as our recent findings in brain tumor animal models show that the therapeutic potential of the restricted KD, involving reduced glucose and elevated β-OHB, is likely to be greater than that for any current brain tumor chemotherapy [20]. Moreover, the KD would eliminate or reduce the need for adjuvant anticonvulsant and steroidal medications for brain tumor patients as the KD has antiepileptic and anticonvulsant effects and, when restricted in caloric intake, will naturally elevate circulating glucocorticoid levels [15,77-80]. These findings suggest that the KD would be an effective multifactorial diet therapy for malignant brain cancer and should be considered as an alternative therapeutic option.

### Dietary Energy Restriction

The findings of the Nebeling group were recently confirmed in a series of orthotopic mouse brain tumor models treated with the KD and dietary energy restriction [21,37,81,82]. As with the KD, dietary energy restriction (DR) reduces glucose and elevates ketone bodies. Indeed, the DR-induced inhibition of brain tumor growth is directly correlated with reduced levels of glucose and elevated levels of ketone bodies [20,21]. This energy transition contributes to the powerful anti-angiogenic effects of DR [81,82]. As a natural dietary therapy, DR improves health, prevents tumor formation, and reduces inflammation [83-88]. DR also improves mitochondrial respiratory function and glutathione redox state in normal cells [87,89]. Thus, DR naturally inhibits glycolysis and tumor growth while enhancing the health and vitality of normal cells and tissues.

The anti-angiogenic effects of DR arise from reduced tumor energy metabolism [21,37,81,82]. This is important since the angiogenic properties of most human gliomas are closely linked to metabolic activity [90]. Previous studies showed that the antitumor effects of DR result from caloric restriction *per se *and not from the restriction of any specific dietary component such as proteins, vitamins, minerals, fats, or carbohydrates [21,88,91,92]. DR or fasting can also reduce cerebral blood flow and oxygen consumption that would further stress brain tumor cells already weakened from reduced glucose levels [33]. Besides reducing angiogenesis, DR also increases brain tumor apoptosis [81,82]. The proapoptotic effects of DR occur in large part from reduced glycolytic energy that most tumors rely upon for growth.

A reduction in glycolytic energy would reduce lactate levels and hydroxyl radical production. This is important since lactate and hydroxyl radicals enhance tumor inflammation as well as cytokine production (tumor necrosis factor α, interleukin-6, and -1β) through glial activation (microglia and astroglia) [93,94]. DR also reduces inflammation and the inflammatory properties of macrophages, while enhancing their phagocytic activities [86,95]. An uncoupling of the detrimental inflammatory properties of tumor associated macrophages from their beneficial phagocytic properties (to remove tumor cell corpses) is considered essential for the eventual management of brain cancer [8]. Hence diet therapies, which lower glucose availability and elevate ketone bodies, can reduce brain tumor growth through integrated anti-inflammatory, anti-angiogenic and pro-apoptotic mechanisms.

## Metabolic Control of Brain Cancer: An Evolutionary Perspective

Based on the differences in energy metabolism between normal brain cells and brain tumor cells, we propose a radically different approach to brain cancer management that combines metabolic control analysis with the evolutionarily conserved capacity of normal cells to survive extreme shifts in physiological environment. The adaptation to environmental extremes is conserved within the genome according to the ecological instability theory of Potts [96]. Consequently, only those cells with a flexible genome will be capable of surviving abrupt changes in metabolic landscape. Cells with genomic defects, which would limit flexibility, should be less adaptable to metabolic stress and therefore vulnerable to elimination through principles of metabolic control analysis. This strategy focuses more on the genetic capabilities of normal cells than on the genetic defects of tumor cells.

As a metabolic disorder involving the dysregulation of glycolysis and respiration, brain cancer is potentially manageable through abrupt changes in metabolic environment. Significant cellular energy is used to maintain the activity of transmembrane ion pumps (the Na^+^, K^+^-ATPase and Ca^2+ ^and Mg^2+ ^ATPases) [29,97,98] (Figure 1). The amount of energy needed to maintain pump function is also greater than that needed for mitosis, which is largely dependent on glycolytic energy [70,97]. Despite differences in membrane potential, most cells have a constant ΔG' of about -57 kJ/mol [14]. According to Veech this is "the still point in the turning world" and if cells cannot maintain this useable ATP they lose potassium, gain sodium and calcium, swell, and eventually die [16]. In other words, regardless of whether the cell is a normal neuron, a glial cell, or even a transformed tumor cell, its survival depends on maintaining an adequate ΔG' of ATP hydrolysis. Tumor cells with limited genomic flexibility should therefore be less capable than normal cells in utilizing alternative energy substrates to maintain their ΔG' of ATP hydrolysis.

The energy used to maintain pump function and cell viability in normal brain cells comes from either glycolysis or aerobic respiration [29,99]. In the case of C6 glioma cells and most brain tumors for that mater, this energy is mostly derived from glycolysis [29,99]. This would then render brain tumor cells vulnerable to reductions in circulating glucose levels as these mutant cells would have difficulty oxidizing alternative fuels (ketone bodies) through respiration. While some brain tumor cells may survive through up-regulation of their glucose transporters [100,101], most will either perish or reduce their growth. Direct support for this hypothesis comes from our recent findings in experimental mouse brain tumor models and from those of Nebeling and co-workers in human pediatric astrocytoma [20,37,75,81].

The widely held notion that brain tumor cells are somehow hardy or tough and resistant to death (programmed or nonprogrammed) may be a misconception. How can brain tumor cells, or any tumor cell for that matter, that have multiple types and kinds of genetic mutations be more fit and hardy than normal cells that possess a flexible genome and can easily transition between glycolysis and respiration for energy maintenance? The notion that tumor cells are more versatile than nontumor cells is also illogical in the context of evolutionary biology. While knowledge of tumor-associated mutations and genomic instability is of considerable academic interest, this information has produced no new or effective clinical therapies for brain tumors. Regardless of when or how genomic defects become involved in the initiation or progression of brain tumors, these defects can be exploited for the metabolic destruction of the tumor.

Normal cells evolved to survive extremes in metabolic and physiological environment due largely to oscillatory changes in the physical environment [96]. Moreover, the ability to adapt to extreme environmental stress is retained within the normal flexible genome [19,102]. It is this flexibility that allows normal brain cells to transition from glucose to β-OHB for energy under reduced energy availability. Due to accumulated nuclear genetic mutations and respiratory defects, brain tumor cells will be less adaptable than normal cells to abrupt changes in metabolic environment and can be either destroyed outright or isolated metabolically from normal cells. Hence, the genomic and metabolic flexibility of normal brain cells can be used to target indirectly the genetically defective and less metabolically flexible brain tumor cells.

According to this hypothesis, a novel strategy can be suggested for human brain tumor management that enhances the respiratory potential of normal brain cells while metabolically targeting the tumor cells. The approach would involve a sequential series of therapeutic steps and should be effective against any primary or secondary brain tumor regardless of cell of origin, anatomical location, or histological grade. Step one would lower circulating glucose levels and elevate circulating β-OHB levels through diet therapies or ketone body supplementation. Glucose ranges between 3.0–3.5 mM (55–65 mg/dl) and β-OHB ranges between 4–5 mM should be effective for tumor management in most patients. The conditions for these parameters have been described previously for children and adults and can be adjusted on a case-by-case basis [34,35,75,103]. These values are also well within normal physiological ranges and will have antiangiogenic and proapoptotic effects causing metabolic isolation and significant tumor shrinkage that can be assessed from imaging analysis. Reduced glucose and elevated ketones could also antagonize tumor cachexia as previously mentioned [82].

Step two would involve surgical resection if necessary. Smaller brain tumors with reduced vascularity and clearly circumscribed boundaries should be easier to resect than larger brain tumors with poorly circumscribed boundaries and extensive vascularization [104]. The diet therapy could also be adjusted following surgery to facilitate healing and to maintain metabolic pressure on any surviving tumor cells.

Finally, step three could involve the use of either conventional or novel targeted therapies. While these therapeutic approaches might have little if any long-term benefit on malignant brain tumor management if used initially, they could be highly effective following the step one strategy after the tumor cells are weakened and metabolically isolated from the physiologically strengthened normal brain cells. Moreover, glycolysis inhibitors that would adversely affect normal cells might also be more effective following the first two steps of the proposed therapeutic strategy [105-109]. It is also possible that carefully executed weight cycling strategies could maintain metabolic pressure on surviving tumor cells and facilitate their eradication or growth retardation [110]. In addition to global diet therapies, more specific amino acid restrictions could also be effective in eliminating surviving tumor cells [111].

The objective of this new brain tumor therapeutic approach is to consistently change the physiological and metabolic environment of the tumor and the host. Only those cells with a normal flexible genome, honed through millions of years of environmental forcing and variability selection, are expected to survive extreme shifts in metabolic environment. Indeed, extreme conditions of survival and fitness will test the limits of a cell population's persistence in any given location over time [96]. While some brain tumor cells might survive under one restricted environment or another, it is unlikely that all tumor cells will survive all restricted environments. In other words, it is the theory of Potts applied with sustained pressure to the entire population of normal and neoplastic brain cells. We predict that this therapeutic approach will be more successful than current approaches because it is based on the principles of evolutionary biology and metabolic control analysis.

## Abbreviations

KD, ketogenic diet; SCOT, succinyl-CoA-acetoacetate-CoA transferase; β-OHB, β-hydroxybutyrate; TCA, tricarboxylic acid.
